# How to discriminate wood of CITES-listed tree species from their look-alikes: using an attention mechanism with the ResNet model on an enhanced macroscopic image dataset

**DOI:** 10.3389/fpls.2024.1368885

**Published:** 2024-06-28

**Authors:** Shoujia Liu, Chang Zheng, Jiajun Wang, Yang Lu, Jie Yao, Zhiyuan Zou, Yafang Yin, Tuo He

**Affiliations:** ^1^ Department of Wood Anatomy and Utilization, Research Institute of Wood Industry, Chinese Academy of Forestry, Beijing, China; ^2^ Wood Collections, Chinese Academy of Forestry, Beijing, China; ^3^ National Centre for Archaeology, Beijing, China; ^4^ Beijing Information Science and Technology University, Beijing, China; ^5^ Wildlife Conservation Monitoring Center, National Forestry and Grassland Administration, Beijing, China

**Keywords:** wood identification, CITES, convolutional neural network, attention mechanism, data enhancement, macroscopic images

## Abstract

**Introduction:**

Global illegal trade in timbers is a major cause of the loss of tree species diversity. The Convention on International Trade in Endangered Species of Wild Fauna and Flora (CITES) has been developed to combat the illegal international timber trade. Its implementation relies on accurate wood identification techniques for field screening. However, meeting the demand for timber field screening at the species level using the traditional wood identification method depending on wood anatomy is complicated, time-consuming, and challenging for enforcement officials who did not major in wood science.

**Methods:**

This study constructed a CITES-28 macroscopic image dataset, including 9,437 original images of 279 xylarium wood specimens from 14 CITES-listed commonly traded tree species and 14 look-alike species. We evaluated a suitable wood image preprocessing method and developed a highly effective computer vision classification model, SE-ResNet, on the enhanced image dataset. The model incorporated attention mechanism modules [squeeze-and-excitation networks (SENet)] into a convolutional neural network (ResNet) to identify 28 wood species.

**Results:**

The results showed that the SE-ResNet model achieved a remarkable 99.65% accuracy. Additionally, image cropping and rotation were proven effective image preprocessing methods for data enhancement. This study also conducted real-world identification using images of new specimens from the timber market to test the model and achieved 82.3% accuracy.

**Conclusion:**

This study presents a convolutional neural network model coupled with the SENet module to discriminate CITES-listed species with their look-alikes and investigates a standard guideline for enhancing wood transverse image data, providing a practical computer vision method tool to protect endangered tree species and highlighting its substantial potential for CITES implementation.

## 1 Introduction

As an integral ecosystem component, trees assume a pivotal role in purifying our atmosphere, serving as habitats for diverse organisms and actively combating climate change (Rivers, 2017). In the intricate fabric of Earth’s ecological system, they are indispensable entities, ensuring the vitality and equilibrium of our planet’s ecological balance. When a tree is harvested, the wood can be used in every stage of human society, such as papermaking, construction, and furniture manufacturing (Falk, 2009; Latib et al., 2020). Approximately 73,000 tree species exist worldwide, but excessive logging and timber over-exploitation have resulted in up to 30% of the world’s tree species being at risk of extinction, along with biodiversity destruction, soil erosion, and other ecological problems (Brancalion et al., 2018; Cazzolla et al., 2022; IUCN, 2022). This threat is true for some tropical tree species, which are widely used in producing high-value furniture, musical instruments, and handicrafts due to their excellent physical processing properties and beautiful patterns (Wick, 2019; Atikah et al., 2021). Thus, driven by high profits, some tree species are being over-harvested, which has been recorded as extinct/extinct in the wild, i.e., *Lachanodes arborea* (Lambdon and Ellick, 2016). Benefits from illegal logging are estimated to account for 15%–30% of the international timber trade, accounting for $51 to $152 billion a year (INTERPOL, 2021).

In recent decades, the world has witnessed a distressing decline in global tree populations. To prevent international trade from threatening the survival of endangered wildlife, the Convention on International Trade in Endangered Species of Wild Fauna and Flora (CITES) came into effect on 1 July 1975 to ensure that international trade in wild animal and plant specimens does not threaten species survival by subjecting international trade in selected species to certain controls (Appendices I, II, and III), according to their needed degree of protection (Goldsmith, 1978). In November 2022, the 19th Conference of the Parties to the CITES was held in Panama, and numerous tropical tree species were newly listed in CITES appendices. To date, more than 34,310 plant species of 134 genera have been included in the CITES appendices, including approximately 670 tree species, and 80% of these species are internationally traded for their timbers (CITES, 2023). Thus, a fast and accurate wood identification method is needed to support CITES implementation and promote legal logging.

Wood, or secondary xylem, is composed of countless cells of different shapes, sizes, and arrangements, with a complex and anisotropic structure (Shmulsky and Jones, 2019). Species within the same genus has similar appearance and even wood anatomical structure. Traditional wood identification, the most mainstream recognition approach, relies on human examination of the anatomical features of wood samples and refers to an identification standard list of the macroscopic and microscopic characters compiled by the International Association of Wood Anatomists (IAWA) (Committee, 1989; Yin et al., 2022). This task can only be completed accurately by experienced wood anatomists who familiarize with wood anatomy and identification, with the help of identification tools and reference materials. From a general point of view, it is arduous for the traditional wood identification method to reach species-level discrimination by observing anatomical features. To compensate for the shortcomings of traditional wood identification methods and break through the wood identification bottleneck at the species level, some techniques, i.e., DNA barcoding (Jiao et al., 2020), mass spectrometry (Deklerck et al., 2019; Price et al., 2021), and near-infrared spectroscopy (Bergo et al., 2016; Pan et al., 2021), have been developed. However, the lack of reference data and the high cost to establish it have limited applications of these approaches.

Computer vision is an interdisciplinary field at the intersection of computer science and image processing that aims to bridge the gap between human visual perception and machines by endowing computers with the capacity to understand, interpret, and extract knowledge from digital visual data (Liu et al., 2017). Image classification is a fundamental computer vision task that attempts to comprehend an entire image to classify images by assigning them to a specific label. With the rapid development of computer vision research and computer hardware performance, several neural network architectures have been proposed for image classification (Wang et al., 2019). Advancements in deep learning and convolutional neural networks (CNNs) have enabled more accurate and robust object and pattern identification with visual data. It is also very appealing to many wood anatomists and has been widely used in wood classification. The automated wood identification method combines deep learning and computer vision to extract structural features and detect key information hidden in wood images (Voulodimos et al., 2019; Hwang and Sugiyama, 2021).

CNNs are the most commonly used computer vision-based wood identification models. As research continues, an array of neural network architectures, including distinguished models such as LeNet, AlexNet, ResNet, and GoogLeNet, have been introduced to address the wood image classification task (Kwon et al., 2017; Ravindran et al., 2018; Oktaria et al., 2019). The performance of these convolutional architectures is boosted by increasing their depth while maintaining their gradient information. In several studies, ResNet has shown superior classification performance compared to other models (He et al., 2021; Wu et al., 2021). However, a wood image is a fine-grained texture image with the characteristics of large intraclass variation and small interclass variation. To accurately determine the wood species, spatial and channel information should be exploited more delicately. Recently, the squeeze-and-excitation network (SENet) was proposed to provide the unit with a mechanism to explicitly model dynamic, nonlinear dependencies between channels using global information (Hu et al., 2018). This mechanism enables the network to selectively amplify or suppress specific feature maps, improving model performance. In this way, SENet can be embedded in the CNN, and the model can be trained to achieve better results.

In many cases, obtaining wood images from reliable sources is difficult, especially for globally regulated tree species, resulting in an unsatisfactory number of high-quality images to satisfy the modeling requirements. Therefore, in the case of limited wood image datasets, transfer learning and data augmentation are effective methods for solving the problem of insufficient data volume (Dyk and Meng, 2001; Ravindran et al., 2018). Kırbaş and Çifci (2022) compared the impacts of several deep learning architectures, namely, ResNet-50, Inception V3, Xception, and VGG19, based on the WOOD-AUTH dataset. They found that Xception performs remarkably well in the transfer learning domain. Hengshuo et al. proposed a wood identification algorithm based on an improved residual CNN, which augments the data based on the self-similarity of wood cross-sectional macrostructure and uses an improved residual CNN model, i.e., ResNet101, based on block gradient weighting to extract the features of each sub-image (Su et al., 2021).

Although there have been many wood species identification studies based on computer vision (Ravindran et al., 2018; Ravindran and Wiedenhoeft, 2020), two main factors still limit the development and application of this technique: the self-collected wood image data from different sources before use have not been adequately processed, and the identification accuracy is mainly affected by the sensitivity of the model to the slight variability in the wood species. Room for improvement in the models used in the existing studies remains.

This study aimed to develop a fast and reliable computer vision-based deep learning model by exploiting spatial and channel information to discriminate CITES-listed tree species from their look-alikes. The specific aims of this study were to (1) construct a CITES-28 (14 commonly traded CITES-listed tree species and 14 of their look-alikes) wood transverse surface image dataset, while concurrently investigating the optimal image data processing approach from the perspective of data enhancement; (2) establish a state-of-the-art SE-ResNet model by embedding the SENet module in a CNN (ResNet); and (3) discriminate CITES-listed tree species from their look-alikes using SE-ResNet in real-world identification.

## 2 Materials and methods

### 2.1 Data preparation and augmentation

In this section, we first explain how to prepare and enhance the wood image data in the experimental preparation stage from the perspective of data enhancement. Second, based on the enhanced data, we select the currently commonly used CNN for model training. Finally, the model performance is evaluated for tree species identification.

#### 2.1.1 Image dataset collection

**Figure 1:**
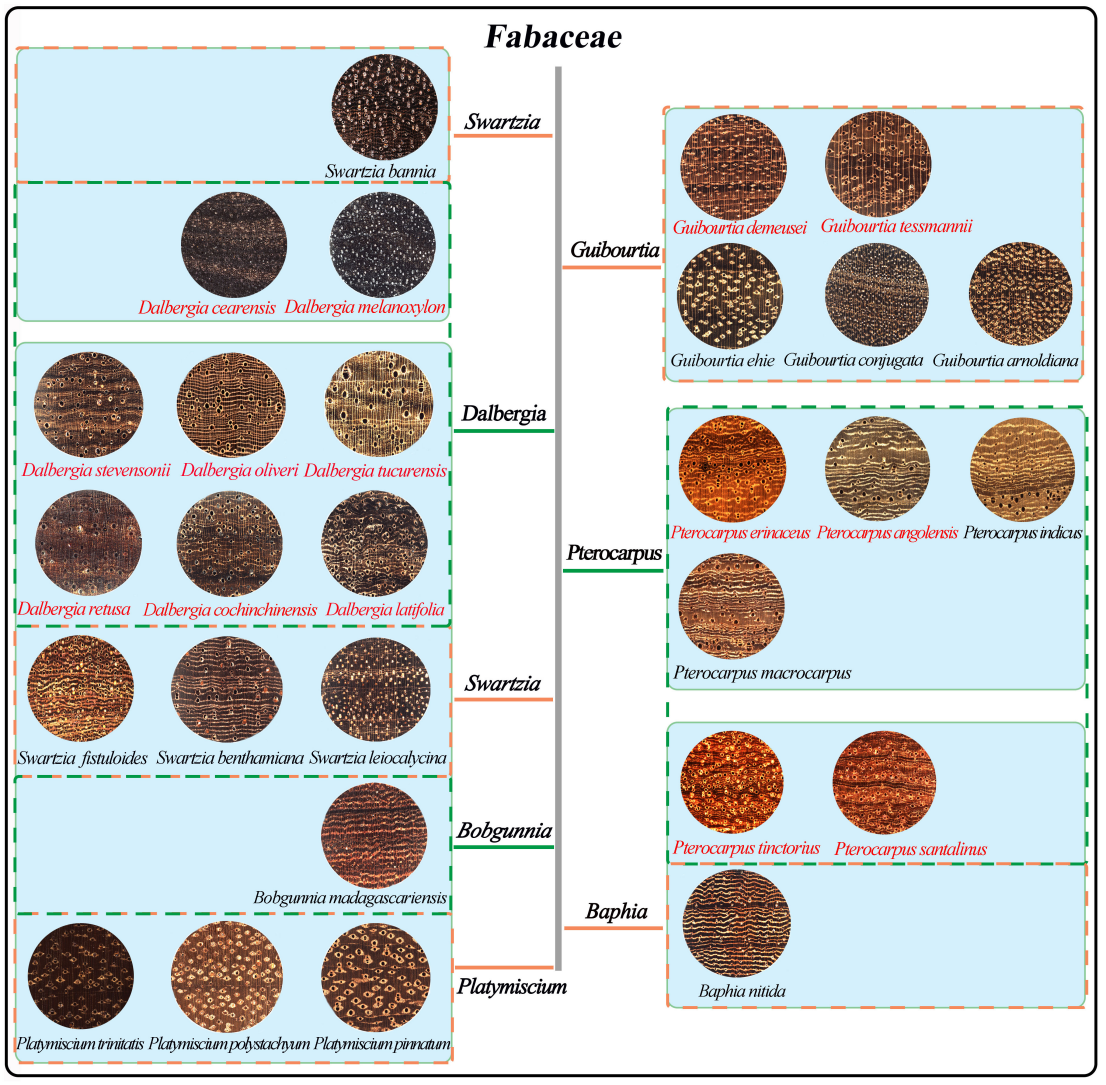
The transverse section of wood species in this research. The CITES-listed wood species and their look-alikes in the same blue frame are often confused (species in red text are CITES-listed). Species in the same dashed box are in the same genus.

In this study, 279 verified specimens of 14 commercially important CITES-listed species and 14 of their look-alike species that were often mixed with CITES-listed species in trade were collected from the Wood Collection of the Chinese Academy of Forestry (CAFw), the USDA Forest Products Laboratory Wood Collection of Madison (MADw), and the Samuel J. Record Collection (SJRw). The transverse surfaces of the wood samples were sanded at grits of 180, 240, 400, 800, and 1,000 to obtain a clear surface for image acquisition. Their macroscopic transverse images are shown in **Figure 1**. The nonoverlapping images of 2,048 × 2,048 pixels, representing 6.35 × 6.35 mm of tissue, were taken with a XyloTron (Ravindran et al., 2020). A total of 9,437 original images were collected to build a CITES-28 dataset of 14 CITES-listed tree species and 14 of their look-alikes based on previous studies (**Table 1**) (Ravindran et al., 2018; Yin et al., 2022).

**Table 1:** Detailed information of the CITES-28 image dataset.

Label	Species (class)	Protection level	Number of collected images	Number of xylarium specimens	Number of images/xylarium specimens
0	*Dalbergia cearensis*	CITES II	321	14	23
1	*Dalbergia cochinchinensis*	CITES II	263	7	38
2	*Dalbergia latifolia*	CITES II	762	21	36
3	*Dalbergia melanoxylon*	CITES II	313	11	28
4	*Dalbergia oliveri*	CITES II	414	6	69
5	*Dalbergia retusa*	CITES II	698	16	44
6	*Dalbergia stevensonii*	CITES II	498	11	45
7	*Dalbergia* *tucurensis*	CITES II	461	12	38
8	*Platymiscium pinnatum*	/	201	10	20
9	*Platymiscium polystachyum*	/	193	16	12
10	*Platymiscium trinitatis*	/	134	6	22
11	*Swartzia bannia*	/	122	5	24
12	*Swartzia benthamiana*	/	106	6	18
13	*Swartzia fistuloides*	/	111	4	28
14	*Swartzia leiocalycina*	/	115	7	16
15	*Bobgunnia madgascariensis*	/	110	5	22
16	*Pterocarpus erinaceus*	CITES II	373	9	41
17	*Pterocarpus santalinus*	CITES II	196	4	49
18	*Pterocarpus tinctorius*	CITES II	263	6	44
19	*Pterocarpus angolensis*	CITES II	805	19	42
20	*Pterocarpus indicus*	/	1,163	29	40
21	*Pterocarpus macrocarpus*	/	600	18	33
22	*Baphia nitida*	/	123	5	25
23	*Guibourtia demeusei*	CITES II	154	4	39
24	*Guibourtia tessmannii*	CITES II	249	7	36
25	*Guibourtia arnoldiana*	/	294	9	33
26	*Guibourtia ehie*	/	337	9	37
27	*Guibourtia conjugata*	/	58	3	19
	**Total**		**9,437**	**279**	~34

The meaning of the symbol "/" represents the species are not protected by CITES.

The bold values represents the total for each column.

#### 2.1.2 Dataset partitioning and patch dataset creation

We divided the dataset before creating patches to avoid dividing the same image into both the training and testing sets. The CITES-28 dataset of the original 9,437 images was divided into 80%/10%/10% training/validation/test splits at the image level. To ensure that the errors are representative of the entire dataset, 10-fold cross-validation for each model was used and accuracy is reported as the average over the 10 folds. An image dataset with a high imbalance results in poor classification performance; hence, six kinds of patches were extracted from the CITES-28 dataset images in this study. More overlap exists in patches of classes (species) with low quantities of images to maintain a balanced distribution of classes (species) in our dataset. The details of the patch dataset used for training and testing are listed in **Table 2**.

**Table 2:** Prediction accuracies of two established models.

Model	Rotation mode	Image compensation	Accuracy (%)
SE-ResNet	Fixed	Y	**99.65**
	Fixed	N	99.55
	Random	Y	99.63
	Random	N	99.47
ResNet	Fixed	Y	**99.45**
	Fixed	N	99.41
	Random	Y	99.28
	Random	N	99.25

Y represents use 0 as a compensation item to complete the processed image.

N denotes that the rotated image is not processed.

The bold values represent the highest accuracy of SE-ResNet and ResNet.

#### 2.1.3 Image turning

We constructed a specific data augmentation method to fully train the model according to the data characteristics, as shown in Equation 1.


(1) aug(xi) = R(xi) + C

where *aug* is the enhancement method to be passed by the sample input to the model training. *R* is the image operation method for wood pictures proposed in this paper. During operation, image samples smaller than the input requirements of the model may be generated; thus, this paper introduces compensation factor *C* to compensate for the phenomenon of missing samples caused by the data enhancement operation.

In the actual test application process, controlling the acquisition angle of the test equipment and other factors is difficult. Therefore, we design a rule-based data enhancement rule with rotation as the core when building the data enhancement method. In addition, considering that the test angle accepted by the test equipment cannot be guaranteed in the actual test process, we propose a random rotation strategy to expand the diversity of training data further. The data enhancement operation can be represented by Equation 2.


(2) $$R\;=\;R_{random}\cap R_{rule}$$

where *R_random_* represents the random rotation strategy and *R_rule_* represents the rule rotation strategy.

**Figure 2:**
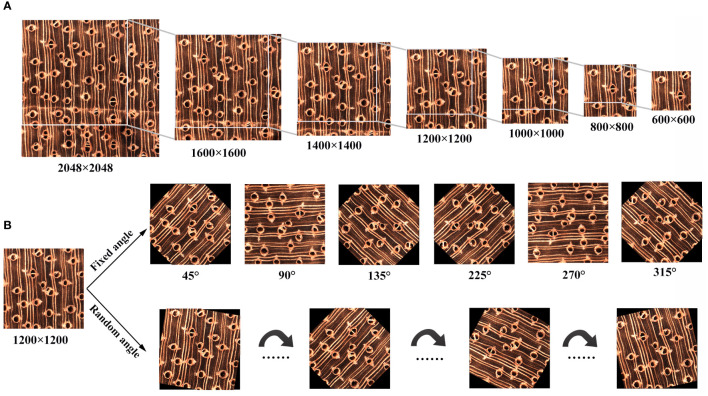
Data augmentation process. **(A)** Patch creation diagram. **(B)** Image rotation and compensation.

The data enhancement process is shown in **Figure 2**. When we enhanced the data based on fixed rotation, the image was rotated to the center with rotation angles of {45°, 90°, 135°, 225°, 270°, 315°}. When we enhanced the data based on a random rotation strategy, the image was rotated to the center, and the rotation angle was randomly sampled between 0° and 360°. After obtaining the rotated image, to meet the requirements that the model can input only the square image region, we cut the rotated image. We used 0 (black) as a compensation item to make up the exact area.

### 2.2 Architecture and training of convolutional neural networks

#### 2.2.1 Convolutional neural network architecture

Based on the enhanced data, we selected ResNet, a widely used network, as the backbone. As He et al. (2016) discovered, a multilayer deep neural network can produce unexpected results, and the training accuracy drops as the layers increase, which is technically known as vanishing gradients. To address this problem, ResNet was proposed to help build a deeper neural network by utilizing skip connections or shortcuts to jump over some layers. SENet, as a classical attention mechanism, can be embedded in the CNN. In this study, we selected SE-ResNet to carry out our experiments.

**Figure 3:**
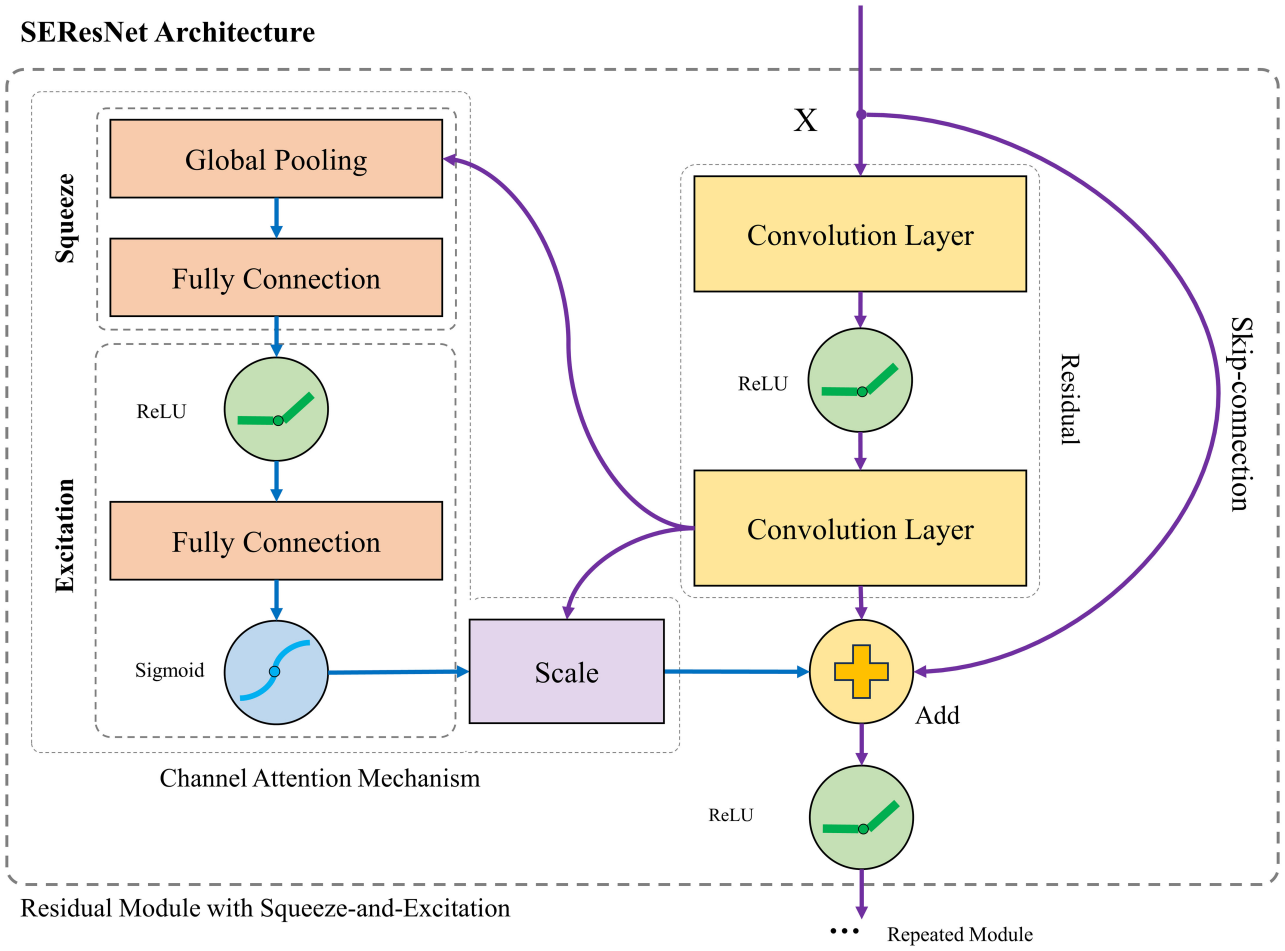
The schematic of the CNN architecture comprises a ResNet with an SENet employed for wood identification.

To better obtain the feature expression of wood images in neural networks, more detailed analysis and processing were carried out on the features among image channels and the depth extraction of two-dimensional features. SENet provides the unit with a mechanism to explicitly model dynamic, nonlinear dependencies between channels using global information (Hu et al., 2018). SENet can ease the learning process and significantly enhance the representational power of the network. The SE-ResNet module includes a block, global average pooling, a fully connected (FC) layer, and an activation function layer with ReLU and sigmoid. The channel attention module is added after the residual module. The schematic architecture of the SE-ResNet model is shown in **Figure 3**.

Equation 3 shows that SENet mines and analyzes the weight information of different channels by studying the relationship between channels. First, it squeezes global spatial information into a channel descriptor by using global average pooling to generate channelwise statistics, which is ρ in Equation 3. Then, it learns to recalibrate the feature adaptively through two FC layers and uses two activation functions to learn nonmutually exclusive relationships. After obtaining the scalars, channelwise multiplication between the scalar and the feature map is carried out to obtain the final output.


(3) $$s=\sigma (W_{2}\delta (W_{1}\rho (x)))$$

#### 2.2.2 Optimization objective

We complete the forward pass and update the network by backpropagation. Globally, the loss function over iterations is still minimized until the loss converges, as shown in Equation 4, where θ is the model parameter, X is the model input, and Y is the corresponding label.

Given an augmented selection, the model is optimized using perturbed features. The feature extraction ability is enhanced during the training process to obtain outstanding performance. In this sense, the augmentations help the model acquire more knowledge of potential input samples, increasing classification performance for real scenes.


(4) $$\hat{\theta }=argminL_{\theta }(\theta |aug(X),Y)$$

### 2.3 Model training

Model training is carried out in two phases. In the first phase, the model pretrained on the ImageNet dataset is studied for image classification via transfer learning. In the second phase, the model is trained to build classifiers for wood identification with the training set and tested at every epoch with the validation set. The initial learning rate of the model is 0.001, the momentum value is set to 0.9, and 24 epochs are trained sequentially. The epoch with the best result in the test set is taken to save the model. Stochastic gradient descent (SGD) is used to optimize the model. The model trained with the following hardware specifications: CPU Intel Core i9-14900K 6.0 GHz, 24 GB of RAM NVIDIA RTX 4090, and 96 GB of GPU. The experimental software environment is Ubuntu 20.04, Python 3.7.13, PyTorch 1.12.1, and NVIDIA CUDA 10.2.

In this study, the following process investigated the optimal image processing method (image rotation mode and patch size) for model accuracy. First, the model was trained with six different image patches extracted from the original image dataset (600 pixels × 600 pixels, 800 pixels × 800 pixels, 1,000 pixels × 1,000 pixels, 1,200 pixels × 1,200 pixels, 1,400 pixels × 1,400 pixels, and 1,600 × 1,600 pixels) to determine the most appropriate size. For the most appropriate patch size dataset, we rotated the patches with two strategies, fixed rotation and random rotation, to achieve the expansion of the image dataset. Compensated or uncompensated processing was performed for images obtained with different rotations. Then, image datasets obtained from different processing strategies were used to train the model and compare the impact of different data enhancement methods on model wood identification performance.

### 2.4 Evaluation

The performance of the trained models was evaluated using a test set. The highest identification accuracy of all models based on test set images is reported. A confusion matrix is given to better understand the classification results of wood species given by the model and to analyze the causes of species discrimination errors in terms of wood anatomy. The confusion matrix contains information about the true and predicted values of the classification and reflects the wood species classification results. Accuracy usually describes model performance on all sample categories and is used when all sample categories are equally important. The higher the value is, the better the performance of the classification model. Accuracy is calculated using true positives (TPs), false positives (FPs), true negatives (TNs), and false negatives (FNs), which are shown in Equation 5.


(5) $$Accuracy\;(\text{A})=\;\frac{TN\;+\;TP}{TN\;+\;TP\;+\;FP\;+\;FN}$$

To further test the generalization ability of the trained model, for each species, we captured 10 images from each sample purchased from the Guangdong Yuzhu Timber Market to conduct real-world identification.

## 3 Results and discussion

### 3.1 Appropriate image data augmentation for wood species identification models

#### 3.1.1 Image cropping

**Figure 4:**
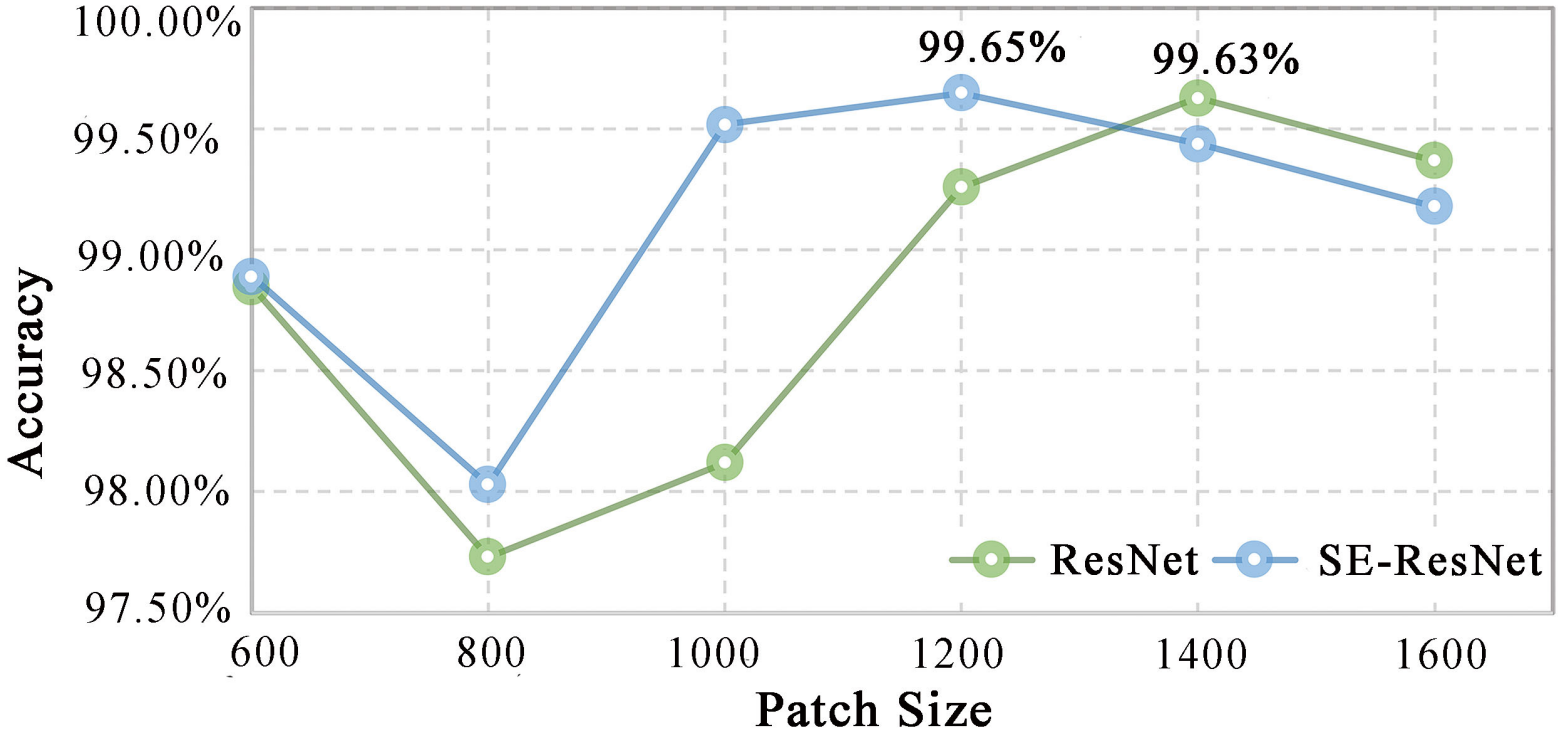
The model performance when using images of different patch sizes.

Cropping is the common means in the present study. **Figure 4** displays the model performance of different patch sizes in this experiment. This figure shows that the ResNet and SE-ResNet models can achieve high identification accuracy when the patch size is larger than 1,200 pixels × 1,200 pixels, which aligns with previous research (He et al., 2020). The appropriate perceptual field of view size is an important factor affecting the classification performance of the model. The computational cost of high-resolution images is too high; thus, the image size must be adjusted but kept within the range where the desired features can be extracted. In addition, information loss should be accounted for when choosing the size of the wood image. For macroscopic images of wood cross-sections, the size of the image feature field of view needs to be balanced with the number of images. Therefore, to obtain as much image data as possible with low overlap while ensuring model accuracy, we consider a patch size of 1,200 pixels × 1,200 pixels to be the optimal size for model training.

#### 3.1.2 Image rotation

**Figure 5:**
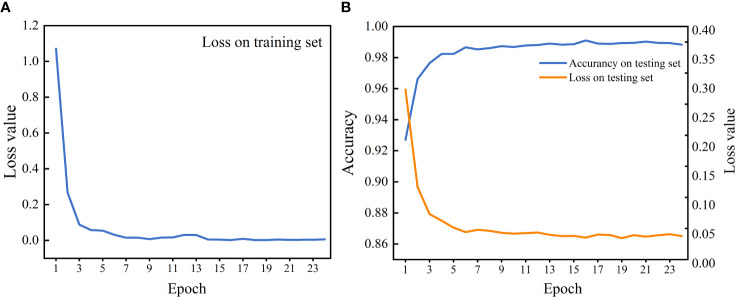
The loss value and accuracy of the SEResNet model across each epoch. **(A)** The model training. **(B)** The model test.

The ResNet and SE-ResNet models were established based on the most suitable patch size. The validity of different image rotation methods on wood identification models was compared for the first time. **Table 3** shows that the fixed rotation method reached higher accuracy for the ResNet (99.65%) and SE-ResNet (99.45%) models. Moreover, these models exhibited relatively high accuracy after using 0 as a compensation item to complete the cropped image; both values were above 99.28%. The results showed that using a fixed rotation method and image compensation to obtain adequate training data is feasible. The accuracy and loss curves of the SEResNet model during the training and test process are shown in **Figure 5**. It showed that learning rate decays by 10 times and tended to be flat when training to 15, respectively. At the same epoch, the test set reached the highest accuracy with a loss value of less than 0.05 (**Figure 5B**). It showed that this model has strong generalization and stability ability and is able to conduct wood species identification.

**Table 3:** Image dataset details with the different patch sizes.

Patch size	Number of train patch	Number of val patch	Number of test patch	Number of total patch
1,600	32,776	3,790	3,889	40,455
1,400	32,157	4,296	4,002	40,455
1,200	32,408	4,029	4,018	40,455
1,000	32,204	3,988	4,263	40,455
800	32,286	3,857	4,312	40,455
600	32,215	4,170	4,070	40,455

Some existing publicly accessible datasets provide numerous images that can be used in computer vision research, but no more attention is given to preprocessing images from different sources before use. Geus et al. (2021) conducted an experiment that applied a rotation of 1° to each image within the training set, covering a range from −15° to 15°. Nevertheless, this experiment did not give specific conclusions about wood image preprocessing methods, and no further research has been carried out. In this experiment, the image undergoes segmentation, followed by rotation over a comprehensive 360° range and subsequent compensation. The results serve to validate the efficacy of the proposed preprocessing approach in substantially expanding the available dataset.

### 3.2 Model accuracy improvement by adding an attention channel

CNN models commonly used in previous studies, such as AlexNet, InceptionV3, and DenseNet, have shown strong feature extraction capabilities when processing two-dimensional images (Geus et al., 2021; Wagle and Harikrishnan, 2021); however, the model performance is unsatisfactory when targeting wood image data. The wood section image contains features with strong regularity. In contrast, the variations between different wood species are subtle. In addition, the difference in wood section images is mainly reflected in factors such as texture, color, and control distribution. The attention mechanism can extract key features of similar images better. In this experiment, the channel attention mechanism is introduced to improve wood species identification accuracy at the species level.

As shown in **Table 3**, the model accuracy of SE-ResNet is generally higher than that of ResNet under various rotation and compensation methods. Compared with ResNet, the performance of SE-ResNet was better and suitable for identifying the CITES-listed wood species with their look-alikes. ResNet incorporates SENet, which enhances the capacity of the network to learn identification keys by adaptively recalibrating the channelwise feature responses. The attention mechanism in computer vision is inspired by the human attention mechanism, which imitates the manner in which people focus more on specific information in an image while ignoring the rest (Lu et al., 2023). As a module that can affect the performance of the model, the attention mechanism can focus limited attention on only the most essential information to save computational resources and obtain the most effective information quickly. In particular, when the original model is underfitting with fewer parameters and cannot fully learn the training data rules, adding the attention module enhances the expressive power of the model, improving the underfitting problem and accuracy.

### 3.3 Discrimination of CITES-listed species from their look-alikes

**Figure 6:**
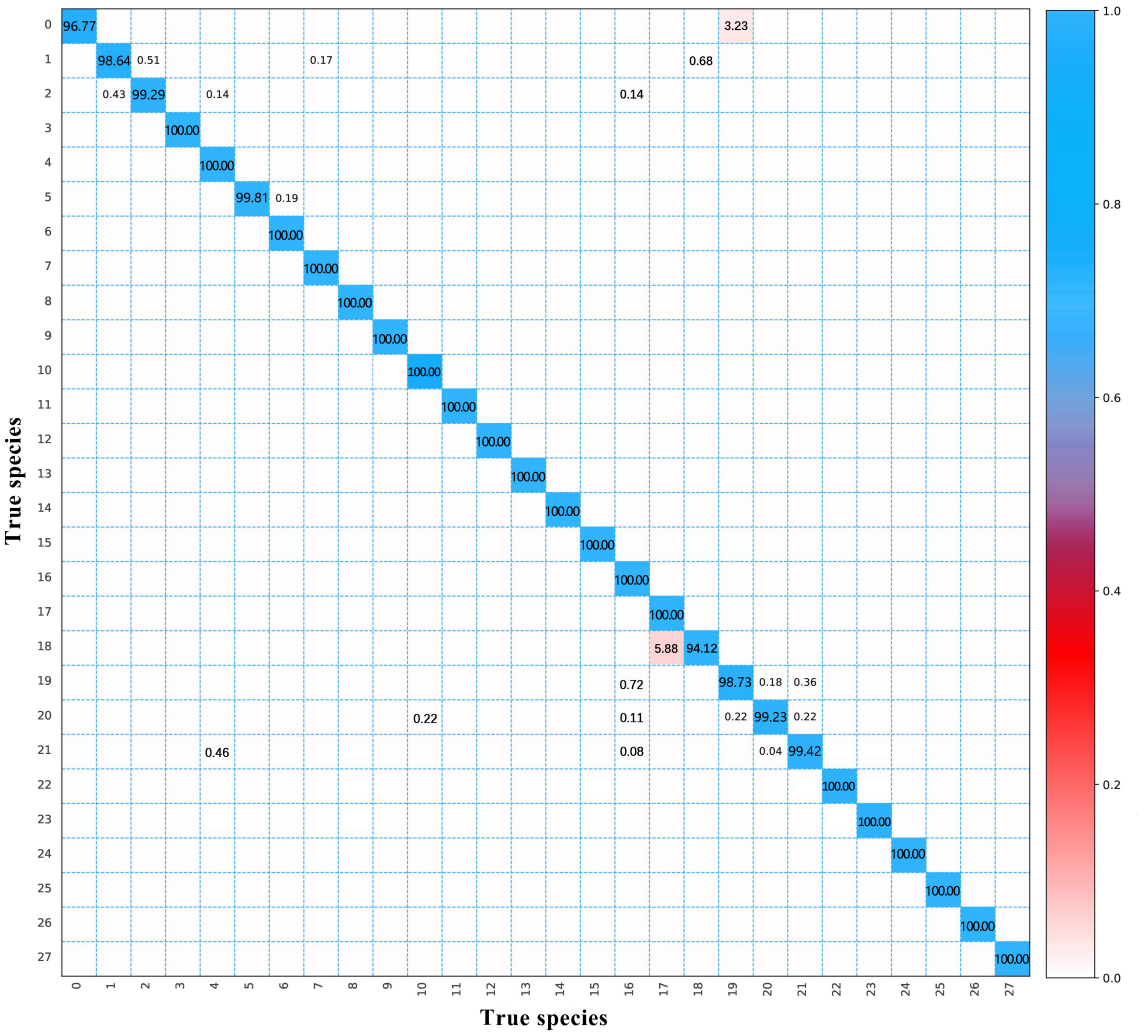
The classification results of the SE-ResNet model with the test dataset. 0—Dalbergia cearensis; 1—Dalbergia cochinchinensis; 2—Dalbergia latifolia; 3—Dalbergia melanoxylon; 4—Dalbergia oliveri; 5—Dalbergia retusa; 6—Dalbergia stevensonii; 7—Dalbergia tucurensis; 8—Platymiscium pinnatum; 9—Platymiscium polystachyum; 10—Platymiscium trinitatis; 11—Swartzia bannia; 12—Swartzia benthamiana; 13—Swartzia fistuloides; 14—Swartzia leiocalycina; 15—Bobgunnia madgascariensis; 16—Pterocarpus erinaceus; 17—Pterocarpus santalinus; 18—Pterocarpus tinctorius; 19—Pterocarpus angolensis; 20—Pterocarpus indicus; 21—Pterocarpus macrocarpus; 22—Baphia nitida; 23—Guibourtia demeusei; 24—Guibourtia tessmannii; 25—Guibourtia arnoldiana; 26—Guibourtia ehie; 27—Guibourtia conjugate.

In this study, the SE-ResNet model obtained the highest accuracy for wood species identification on a dataset with fixed and complementary rotation. The confusion matrix in **Figure 6** shows the classification results of the model for each wood species in the test set. Overall, the classification accuracy of the 28 tree species reached 99.65%, with 20 wood species correctly classified. Surprisingly, the predictions made by the SE-ResNet model for five *Guibourtia* species were perfect. When discriminating CITES-listed *Dalbergia* species, they were identified with an accuracy of over 95% with this model. The CITES-listed *Dalbergia* species was completely distinguished from eight look-alike species, including *Platymiscium*, *Swartzia*, and *Bobgunnia*. However, approximately 3.23% of the *Dalbergia cearensis* images were misclassified as *Pterocarpus angolensis*.

Within *Pterocarpus*, all the CITES-listed *Pterocarpus* species were completely discriminated from their look-alikes, except for *Pterocarpus angolensis*, which was confused with *Pterocarpus indicus* and *Pterocarpus macrocarpus*. For the closely related *Pterocarpus* species, *Pterocarpus angolensis*, *Pterocarpus erinaceus*, *Pterocarpus macrocarpus*, and *Pterocarpus indicus* were misjudgments between each other. These four species have large wood anatomical similarities, such as the axial parenchyma arrangement and ray type, which make them difficult to distinguish completely and accurately on the basis of macroscopic images of a single cross-section. Not surprisingly, *Pterocarpus santalinus* and *Pterocarpus tinctorius* appeared to be misidentified, with 5.88% of the images of *Pterocarpus tinctorius* misclassified as *Pterocarpus santalinus*. With the traditional methods, it is extremely difficult to distinguish between these two species because of their highly similar macroscopic and microscopic structural characteristics. Therefore, *Pterocarpus tinctorius* was listed in CITES Appendix II in 2019 due to its over-exploitation and extreme similarity with *Pterocarpus santalinus*, listed in CITES Appendix II in 2007.

The vast majority of wood and wood products have lost the key identification characteristics, such as leaves and bark, which increases the difficulty of wood identification. In particular, wood species of the same genus or even closely related genera have high similarities in appearance and anatomical characteristics. Not surprisingly, compared to human vision-based methods, the results demonstrated in this experiment can provide higher identification accuracy (Wiedenhoeft et al., 2019). Simultaneously, the deep learning model effectively eradicates human subjectivity and assumes the role of a professional with greater efficiency and precision.

### 3.4 Prospect of wood species identification based on a deep learning model

**Table 4** presents the discriminative results of the SE-ResNet model for images collected from the timber markets. For completely unfamiliar samples, the recognition accuracies of SE-Resnet and Resnet are 82.3% and 70.4%, respectively; the generalization ability of SE-Resnet model is superior. For SE-Resnet, the classification results show that images of 16 species were fully identified and 6 species had 8 or 9 out of 10 images accurately identified. In this regard, the CITES-listed tree species of *Pterocarpus* and *Guibourtia* were all distinguished from their look-alikes. *Dalbergia* spp., *D. cearensis*, and *D. cochinchinensis* were also accurately identified. However, this model performed worse when discriminating *Platymiscium* spp., with images of all three species at a level of poor stability. Although the number of samples is large, the effective area of the image that can be acquired for each sample is small and there is a relatively small number of images of *Platymiscium* used for modeling. Such a phenomenon also appears in *Swartzia benthamiana* with *Swartzia leiocalycina*. It demonstrated that even if more images could be obtained through data enhancement strategies, fully covering the variability within species would not have been possible.

**Table 4:** The identification performance of the models for independent images.

Label	Species (class)	SE-Resnet		ResNet	
		Accuracy%	Misidentified labels	Accuracy%	Misidentified labels
0	*Dalbergia cearensis*	100	/	100	*/*
1	*Dalbergia cochinchinensis*	80	*Dalbergia retusa* *Dalbergia stevensonii*	0	*Pterocarpus santalinus* *Dalbergia latifolia* *Dalbergia stevensonii*
2	*Dalbergia latifolia*	100	/	100	*/*
3	*Dalbergia melanoxylon*	100	/	100	*/*
4	*Dalbergia oliveri*	80	*Dalbergia stevensonii*	100	*/*
5	*Dalbergia retusa*	100	/	80	*Dalbergia oliveri*
6	*Dalbergia stevensonii*	100	/	0	*Dalbergia tucurensis* *Dalbergia retusa*
7	*Dalbergia tucurensis*	80	*Dalbergia stevensonii*	70	*Pterocarpus indicus* *Dalbergia oliveri*
8	*Platymiscium pinnatum*	40	*Platymiscium polystachyum* *Platymiscium trinitatis*	70	*Platymiscium trinitatis*
9	*Platymiscium polystachyum*	20	*Platymiscium pinnatum*	40	*Platymiscium pinnatum*
10	*Platymiscium trinitatis*	20	*Dalbergia melanoxylon* *Dalbergia retusa*	50	*Dalbergia stevensonii Platymiscium polysta*
11	*Swartzia bannia*	100	/	60	*Dalbergia stevensonii* *Dalbergia oliveri*
12	*Swartzia benthamiana*	60	*Dalbergia cochinchinensis* *Dalbergia stevensonii*	70	*Pterocarpus angolensis*
13	*Swartzia fistuloides*	100	/	100	*/*
14	*Swartzia leiocalycina*	40	*Platymiscium trinitatis* *Swartzia fistuloides*	10	*Swartzia bannia* *Platymiscium trinitatis*
15	*Bobgunnia madgascariensis*	100	/	90	*Dalbergia cearensis*
16	*Pterocarpus erinaceus*	100	/	100	*/*
17	*Pterocarpus santalinus*	90	*Pterocarpus macrocarpus*	90	*Dalbergia retusa*
18	*Pterocarpus tinctorius*	90	*Pterocarpus santalinus*	100	*/*
19	*Pterocarpus angolensis*	100	/	100	*/*
20	*Pterocarpus indicus*	100	/	90	*Dalbergia oliveri*
21	*Pterocarpus macrocarpus*	80	*Pterocarpus angolensis* *Pterocarpus indicus*	50	*Dalbergia oliveri*
22	*Baphia nitida*	100	/	100	*/*
23	*Guibourtia demeusei*	100	/	90	*Pterocarpus tinctorius*
24	*Guibourtia tessmannii*	100	/	10	*Guibourtia demeusei*
25	*Guibourtia arnoldiana*	40	*Guibourtia ehie*	0	*Guibourtia ehie*
26	*Guibourtia ehie*	100	/	100	*/*
27	*Guibourtia conjugata*	100	/	100	*/*
	**Total**	**82.3**		**70.4**	

The meaning of the symbol "/" represents the species don’t misidentified as other wood species.

The bold values represents the average accuracy of SE-ResNet and ResNet.

Based on this result, we considered that the model proposed in this experiment could identify 22 tree species accurately and consistently. The SE-ResNet model has great potential for generalization, and the model gives better recognition for wood species with more specimens. Thus, it is recommended that more specimens of the same species be collected to train the model to cover as much variability as possible and that, where possible, many specimens be used to test the generalization ability of the model in future studies, which can enhance the accuracy, generalization ability, and applicability of the trained model.

Our experiment explored the effect of image cropping and rotation on the performance of deep learning models for wood image identification and added an attention channel module into the ResNet-50 model. It was found that fixed rotation and cropping are effective data enhancement methods for wood images. In addition, we added only the attention channel module into the models, and other attention mechanisms, such as multi-head self-attention, self-attention, and convolutional block attention modules, were not tested in wood species identification (Woo et al., 2018; Guo et al., 2022). Follow-up research should explore more data enhancement methods, such as mirroring and scaling. The generative adversarial network (GAN) technique is a new computer vision method. The GAN-based oversampling technique not only increases the minority class representation to solve class imbalance problems but also may help to prevent overfitting (Sampath et al., 2021). However, GANs have not been reported in wood image recognition.

Numerous practical tools have been developed to address the specific field application requirements, exemplified by solutions such as MyWood-ID (Tang et al., 2018) and XyloPhone (Wiedenhoeft, 2020). Considering the comprehensive research conducted in this paper, an intelligent wood identification system was devised and successfully deployed for on-site inspections carried out by customs officials. Compared to other emerging techniques (NIRS, DART-MS, and DNA barcode), the computer vision-based wood identification approach demonstrates considerable promise in achieving species-level precision in field screening for wood species.

At this research stage, the dataset images are mainly processed on the surface of the wood by both knifing and sanding in the laboratory so that the anatomical characteristics of the wood can be fully exposed before the images are acquired. The experimentally acquired images after fine sanding are idealized, and the models trained based on these image data may not match the practical application. Therefore, exploring the deep learning models with wood images of rough surfaces is necessary to reduce the workload of wood surface treatment by processing rough surface images.

## 4 Conclusion

The traditional wood species identification method has a history of nearly a century, forming a complete identification process and norms, and still occupies a dominant position. However, in the new era of demand, traditional methods need to be complemented by new technologies to achieve species-level wood species identification. Faced with the current conservation pressure of endangered tree species, a more accurate, convenient, and economically friendly method is urgently needed for the identification of wood species. Computer vision is the most feasible technique for traded wood species identification coupled with deep learning, especially for the field inspection at import and export ports.

The results of this study showed that the 1,200 pixel × 1,200 pixel patch size can be applied as the best choice for the training model and that the number of wood images can be effectively expanded by image cropping and rotation. In addition, the channel attention mechanism (SENet) module is added to the CNN structure to identify CITES-listed tree species and their look-alikes with an accuracy of over 99%. It shows a relatively satisfactory performance in real-world identification. For the public, the model does not require extensive knowledge of wood anatomy and experience in species identification, which greatly reduces the complexity of the traditional wood identification process.

This work not only provides a CNN model along with added attention channels for successful identification but also provides a standard guideline for image data enhancement when conducting wood species identification. Further studies are needed to explore the interspecific wood anatomy features with deep learning models and feature visualization. The results of this study show that the wood transverse image dataset coupled with the SE-ResNet model can accurately discriminate CITES-listed species from their look-alikes to combat illegal timber trade and contribute to tree species conservation.

## Data availability statement

The original contributions presented in the study are included in the article/supplementary material. Further inquiries can be directed to the corresponding author.

## Author contributions

SL: Data curation, Writing – original draft, Writing – review & editing, Methodology, Visualization. CZ: Methodology, Software, Writing – review & editing. JW: Data curation, Writing – review & editing. YL: Data curation, Writing – review & editing. JY: Methodology, Writing – review & editing. ZZ: Software, Writing – review & editing. YY: Funding acquisition, Writing – review & editing. TH: Funding acquisition, Writing – review & editing.
